# Sex-dimorphic gene expression and ineffective dosage compensation of Z-linked genes in gastrulating chicken embryos

**DOI:** 10.1186/1471-2164-11-13

**Published:** 2010-01-07

**Authors:** Shaobing O Zhang, Sachin Mathur, Gaye Hattem, Olivier Tassy, Olivier Pourquié

**Affiliations:** 1 Stowers Institute for Medical Research, 1000 E. 50th Street, Kansas City, MO 64110, USA; 2 Howard Hughes Medical Institute, Stowers Institute for Medical Research, 1000 E. 50th Street, Kansas City, MO 64110, USA; 3 University of Missouri-Kansas City, School of Computing and Engineering, 5100 Rockhill Road, Kansas City, MO 64110 USA; 4 Department of Anatomy & Cell Biology, The University of Kansas School of Medicine, Kansas City, KS 66103 USA; 5 IGBMC (Institut de Génétique et de Biologie Moléculaire et Cellulaire), Illkirch, F-67400 France; Inserm, U.964, Illkirch, F-67400 France; 6 IGBMC (Institut de Génétique et de Biologie Moléculaire et Cellulaire), Illkirch, F-67400 France; Inserm, U.964, Illkirch, F-67400 France; CNRS, UMR7104, Illkirch, F-67400 France; Université de Strasbourg, Strasbourg, F-67000 France

## Abstract

**Background:**

Considerable progress has been made in our understanding of sex determination and dosage compensation mechanisms in model organisms such as *C. elegans*, *Drosophila *and *M. musculus*. Strikingly, the mechanism involved in sex determination and dosage compensation are very different among these three model organisms. Birds present yet another situation where the heterogametic sex is the female. Sex determination is still poorly understood in birds and few key determinants have so far been identified. In contrast to most other species, dosage compensation of bird sex chromosomal genes appears rather ineffective.

**Results:**

By comparing microarrays from microdissected primitive streak from single chicken embryos, we identified a large number of genes differentially expressed between male and female embryos at a very early stage (Hamburger and Hamilton stage 4), long before any sexual differentiation occurs. Most of these genes are located on the Z chromosome, which indicates that dosage compensation is ineffective in early chicken embryos. Gene ontology analyses, using an enhanced annotation tool for Affymetrix probesets of the chicken genome developed in our laboratory (called Manteia), show that among these male-biased genes found on the Z chromosome, more than 20 genes play a role in sex differentiation.

**Conclusions:**

These results corroborate previous studies demonstrating the rather inefficient dosage compensation for Z chromosome in birds and show that this sexual dimorphism in gene regulation is observed long before the onset of sexual differentiation. These data also suggest a potential role of non-compensated Z-linked genes in somatic sex differentiation in birds.

## Background

Many metazoan species have dimorphic sex chromosomes. The imbalanced or differential expression of sex determination genes on the sex chromosomes of a given species controls the genetic cascade that eventually leads to dimorphic development of reproductive organs and secondary sexual characteristics. Different mechanisms of sex determination have been uncovered in genetically tractable systems such as *C. elegans*, *Drosophila *and the mouse [1]. The majority of genes located on the sex chromosomes, however, are not involved in sex determination. Their imbalanced expression in the two sexes can have deleterious consequences in species like mammals [2]. Thus, various species have evolved different mechanisms to equalize the expression levels (dosage compensation) of these genes.

In mammals, the parity of female and male expression of X genes is achieved through inactivation of one whole X chromosome in the female and upregulation of the single X gene copy in both females and males to equalize the expression level with the autosomes [2]. In the fly, both copies of X chromosomes are active in the female [3]. An increase in the expression of the single X gene copy in the male brings the expression level closer to that of the two female copies and close to that of autosomes. In *C. elegans*, transcription from the two active X gene copies in hermaphrodites is first decreased by one-fold to equal that of a single X gene copy in the male [3]. Then, transcription from the two decreased X gene copies in hermaphrodites and the single copy in the male are further increased to equal that of autosomal copies [3].

The avian species represents an interesting but poorly understood system in which the homogametic karyotype (ZZ) corresponds to the male, while the heterogametic karyotype (ZW) corresponds to the female [4,5]. This is in contrast to mammals or *Drosophila*, in which the homogametic karyotype (XX) represents the female and the heterogametic (XY) represents the male. It is currently unclear whether the male sex is determined by the presence of the two Z chromosomes, or whether the female sex is defined because of the presence of the W chromosome [4,5]. Recently, microarray-based, genome-wide, gene-profiling studies of male and female adult zebra finch and chicken embryos have demonstrated that a majority of genes located on the Z chromosome are not dosage-compensated, and thus are expressed at a much higher level in males than in females [6-12]. The lack of compensation of Z genes could lead to an increase in expression of male determinants in ZZ individuals, ultimately leading to male sex determination. Consistent with this idea, DMRT1, a Z-linked gene was recently shown to be required for male gonadal sex determination [13]. Alternatively, it has been proposed that the PKCIW gene (also known as HINTW), which is located on the W chromosome, is involved in female sex determination [14,15] and inhibits the activity of its homolog on the Z chromosome, PKCIZ. This hypothesis is closer to what is observed in mammalian sex determination in which the male sex is defined by specific male determinants, such as the *sex determining region Y *(*Sry*) gene located on the Y chromosome [16]. The functional significance of PKCIW in female sex determination remains, however, unknown.

Here, we use chicken whole-genome Affymetrix microarrays to compare gene expression profiles of the primitive streak of individual male and female gastrulating chicken embryos at day 1 of incubation. This is a critical stage at which axis formation and neural, ectodermal, mesodermal and endodermal patterning begin. Dosage compensation is observed already at this stage for a small portion of Z-linked genes. Compared to dosage compensation of Z-linked genes as reported during the late stages [8], dosage compensation of Z-linked genes at this stage shares similarities, such as an enrichment at the male hypermethylated (MHM) region, but differs in terms of which genes are compensated. It indicates a temporal regulation of dosage compensation in the chicken. We also report that a large number of genes, which reside mainly on the Z and W chromosomes, are dimorphically expressed between male and female embryos. Surprisingly, functional annotation of the male-biased Z genes revealed significant enrichment in the genes involved in male sex determination/differentiation. We conclude that the lack of Z chromosome-wide dosage compensation may be part of the mechanism of sex differentiation at early stages when there is no obvious morphological difference between sexes.

## Results and Discussion

### Sex-biased gene expression in Hamburger and Hamilton Stage 4 chicken embryos

**Figure 1:**
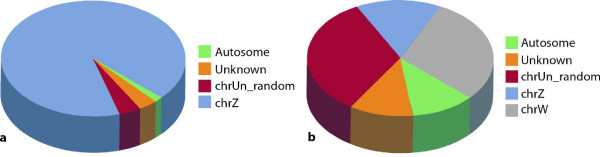
**Chromosomal distribution of dimorphically expressed genes**. (**a**) The distribution of male-biased genes with M:F ratios greater than 1.5. Total probeset number is 275. (**b**) The distribution of female-biased genes with F:M ratios greater than 1.5. Total probeset number is 27.

We analyzed 18 samples dissected from the anterior primitive streak region in 18 independent Hamburger and Hamilton Stage 4 (HH4) [17] chicken embryos. Expression of the female-specific PKCIW gene indicates that nine samples are female, while the other nine, which lacked this expression, are male [15]. Total RNA was extracted, amplified, labeled and hybridized to Affymetrix GeneChip® Chicken Microarrays. Microarray expression data were processed as described in Methods. Only the 14548 probesets that were detected as "Present" in at least nine of the 18 samples were retained for further analysis. Significance Analysis of Microarrays (SAM) test [18] was used to determine which genes were significantly overexpressed in males compared to females. The ratio of mean values of expression of males and females (M:F) of each probeset was also computed. Among the 14548 probesets, 406 probesets (246 genes) are expressed significantly higher in males than in females with a false discovery rate (FDR) of 5% (Additional file 1), while 275 probesets (179 genes) out of the 406 probesets differentially expressed in males have a M:F ratio greater that 1.5 and smaller than 3.2. These 275 probesets were considered, hereafter, as dimorphically male-biased (Additional file 2). Four probesets (three genes) are located on autosomes 1, 2, 10 and 11, and 11 probesets are unassigned on chrUn_random, while nine are of unknown chromosome location. The majority of the dimorphic probesets (251 probesets, 170 genes) are on the Z chromosome (Figure 1a).

When computing the F:M ratios of mean expression levels of the 14548 probesets, 35 were detected as being expressed significantly higher in females with a FDR of 5% (Additional file 3), while 27 out of the 35 probesets differentially expressed in females exhibit a F:M ratio greater than 1.5. These 27 probesets are regarded as dimorphically female-biased (Additional file 4). Three probesets are on chr1, four on chrZ, nine on chrUn_random, three are of unknown location and eight are on chrW (Figure 1b). Since the W chromosome is only partially sequenced and annotated at present, it is likely that at least some of the nine probesets (aligned on chrUn_random) with F:M ratios higher than four are also on the W chromosome. Among these genes, interesting candidates include the transcription factor BTF3, which shows a F:M ratio of 130 and was identified as a transcriptional co-activator for the estrogen receptor [19]. The STAT-related gene NP_001012932.1, which shows a F:M ratio of 7, is also of interest since STAT5 was implicated in sexual dimorphic growth downstream of growth hormones in mammals [20].

An obvious difference between the male- and female-biased/dimorphic probesets is the value of the M:F ratio (mean 1.75) and F:M ratio (mean 95.2). While the male-biased probesets are detected in males at a level ~one-fold higher than in females, the female-biased probesets exhibit a much higher increase. This difference may be explained as the result of a majority of male-biased genes being located on the Z chromosome, which has two gene copies in males and only one copy in females. Among the female-biased genes, those on chrUn_random may be not-yet-annotated genes on W chromosome. If so, the bulk of the female-biased genes would be on W, which has one copy in females but is absent in males. However, the expression of chr1 genes with F:M ratio greater than 2 cannot be attributed to the difference in gene copy number.

### Absence of chromosome-wide compensation of Z-linked genes

**Figure 2:**
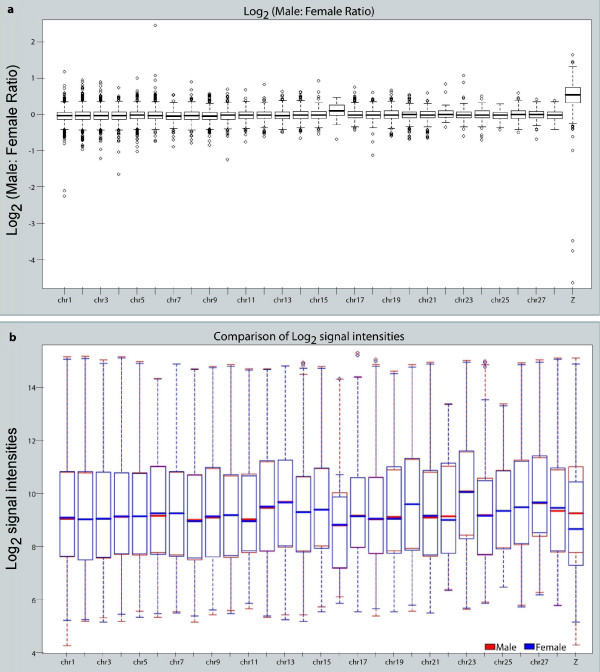
**Comparison of M:F ratios and expression levels of Z-linked genes to those of autosomal genes**. (**a**) The average values of M:F ratios for autosome chr1-chr28, along with that of Z chromosome. Values are represented on log2 scale. (**b**) The distribution of the expression level of genes on autosome chr1 to chr28 and on chrZ in female (blue) and male (red). Values are represented on log2 scale. For simplicity, the W chromosome is not included. Chromosome 32 is not annotated and thus not included in this analysis.

Therefore, it appears that already at the gastrulation stage, male-biased genes are overrepresented on the Z chromosome over autosomes. To determine if this over-representation is statistically significant, we used a Wilcoxon Rank Sum Test on the distribution of log_2 _(M:F) values for genes from autosomes and Z chromosomes. A *p value *of < 2.2e-16 was obtained, which supports the significance of overrepresentation of the male-biased genes on Z. This is consistent with other results [7,9-11]. The mean M:F ratios of all expressed genes on 28 autosomes were plotted together with the mean M:F ratios of expressed Z genes. The mean M:F ratio of autosomes is close to zero on the log_2 _scale, while the mean M:F ratio of Z is 0.51 (Figure 2a). This suggests that the Z chromosome-linked genes are not well equalized/compensated between males and females.

To determine if this M:F ratio of Z genes can be explained by a global increased expression of Z chromosome genes compared to autosomes in males or by a decreased expression on the single Z chromosome in females, the mean expression levels of autosomal genes in comparison with Z genes in both females and males were plotted. In males, the expression level of Z genes is similar to that of autosomes (*P*-value 0.226, Wilcoxon Rank Sum Test). However, in females, there is a significant decrease in the expression of Z genes compared to autosomal genes (*P*-value 1.107e-07, Wilcoxon Rank Sum Test) (Figure 2b). This is in sharp contrast to all three different situations in mammals, flies and worms, and is consistent with the results reported in older embryos [6,7,9], indicating that the high M:F ratio of Z genes is achieved through the relatively low expression of genes from the single copy on Z in females. In other words, dosage compensation between females and males, and between Z chromosome and autosomes, is either nonexistent or ineffective at the chromosome-wide level.

### Regional differences in the distribution of compensated gene expression along the Z chromosome

**Figure 3:**
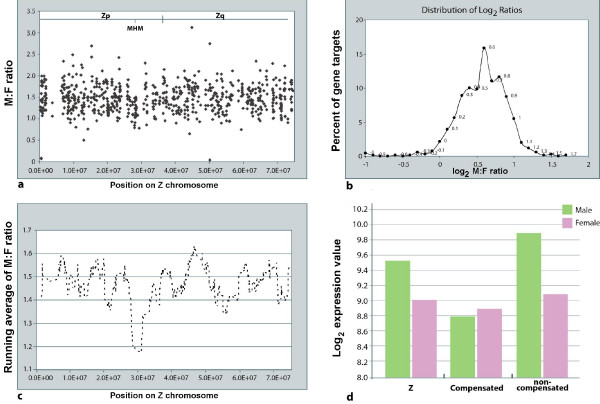
**Distribution of M:F ratios and average expression levels of Z-linked genes**. (**a**) The distribution of M:F ratio (Y axis) as a function of position on Z chromosome (X axis). Zp arm, Zq arm, centromere and MHM region of Z chromosome are indicated. (**b**) The percentile distribution (Y axis) of Z genes as a function of ratio values (X axis, log2 scale). (**c**) The trend of M:F ratio distribution is represented as running averages of 30 consecutive ratios positioned at the median position of the 30 ratios. (**d**) The average expression level (Y axis, log2 scale) of all expressed Z genes (Z), compensated Z genes (Compensated) and non-compensated Z genes along with autosomal genes in females and males.

Not all genes of the Z chromosome show a male-biased expression, raising the question as to what extent dosage compensation occurs in these genes. If dosage compensation occurs for Z genes, ideally, a M:F ratio of 1 is expected. We extracted all Z-linked probesets from the 14548 analyzed. In total, 689 Z-linked probesets (387 genes) are expressed in day 1 embryos. The M:F ratios of these probesets were computed (Additional file 5) and plotted as a function of position along the Z chromosome (Figure 3a). A prominent feature of the M:F ratio pattern is that the ratios are quite heterogeneous. The majority exhibit a value between 1 and 2. This is different to the situation in which all genes are compensated (M:F = 1), and the situation in which all genes are non-compensated (M:F = 2). The fact that the major peak of M:F ratio is centered at 1.52 (log_2 _= 0.6) suggests that dosage compensation in day-1 chicken embryos is not either all or none (Figure 3b) [6,8]. The probesets with ratios of 0.8-1.3 were considered as compensated (Additional file 6), while the probesets with ratios above 1.5 were not (Additional file 7). 189 probesets (114 genes) and 309 probesets (193 genes) were found to be compensated and non-compensated, respectively. Compensated and non-compensated genes are distributed across the whole Z chromosome. The ratios display roughly a bell-shaped distribution pattern with a single peak of 0.6 at log_2 _(ratio) and two shoulders at 0.4 and 0.8, respectively (Figure 3b). The shoulder at 0.4 corresponds to the probesets classified as compensated (M:F ratio 0.8-1.3). The major peak and the other shoulder consist of probesets classified as non-compensated (M:F ratio >= 1.5). Thus, the distribution is trimodal rather than bimodal as observed in the embryonic brain, heart [9] and liver [7] during later embryonic stages. It appears closer to the distribution pattern observed in the embryonic brain [7].

In chicken embryos, dosage compensation with a M:F ratio of expression of 1:1 occurs in a small portion of Z-linked genes that are mostly associated with the male hypermethylated MHM [8,21]. To examine the distribution trend of M:F ratios across the Z chromosome position, the running averages of ratios were calculated as previously described [8]. In agreement with these two studies, a major valley with compensated genes enriched at MHM region was observed (Figure 3c). This region corresponds to a short genomic region located on the Z chromosome, which is hypermethylated in males and, which in females, produces a large non-coding RNA that remains associated with the MHM region. The W chromosome is required for the transcription of the female-specific MHM transcript, supporting the possibility that in the chicken, sex determination requires the W chromosome [21]. The MHM non-coding RNA was shown to be expressed in HH5 to HH6 female chicken embryos [21]. However, there is no probeset in the Affymetrix GeneChip that detects MHM non-coding RNA. Thus, we do not know whether MHM non-coding RNA is already expressed in HH4 chicken embryos.

We compared the results of compensated and non-compensated gene lists to those of Itoh [7] and Melamed [8]. Valley and peak genes from the Melamed *et al*. study were compared with compensated and non-compensated genes in this study, respectively. Thirteen out of 61 Melamed valley genes were shared with the compensated genes and six with non-compensated genes, and 32 of 121 Melamed *et al*. peak genes were shared by non-compensated genes and eight with compensated genes from our study. Many of the peak and valley genes of the Melamed study were not expressed in our dataset (data not shown). Genes with a M:F ratio > 1.5 were extracted from the brain study of Itoh *et al *and compared to the male-biased probesets (see additional data file 2 of Itoh *et al*, 2007). Of the 277 male-biased probesets from Itoh *et al*, 157 (56%) were represented in the male-biased set in our study (data not shown). Interestingly, the major Zq peak centering on position 6.0 × 10^7 ^bp observed in E14 embryonic brain, heart and liver [8] and E10, E15, E19 adult brain and gonad tissues [8] is not observed in our study (Figure 3c). Only 40 of the 121 genes of the peak identified as non-compensated [8] were identified in this analysis. In contrast, a major peak is observed at a different position around 4.6 × 10^7 ^bp on the chromosome arm Zq in our study (Figure 3c). Furthermore, genes distally located to the MHM on Zp are expressed at relatively higher M:F ratios, with multiple peaks in contrast to the levels reported in the brain, heart and liver tissues of E14 embryos (Figure 3c) [8] and E10, E15, E19 adult brain and gonad tissues [8].

Using brain and gonad tissues collected from E10 to adult stages, Mank & Ellegren [11] computed the "amplitude" of gene expression on Z chromosome. They reached the conclusion that Z chromosome dosage compensation does not have enrichment at the MHM region and is not subject to temporal regulation within the same tissue type. This different conclusion was a result of using "amplitude" instead of "M:F ratio" and a different view on whether genes with low (female-biased) M:F ratios should be considered as compensated [22,23]. We computed the same "amplitude" profile of Z-linked genes in addition to the "M:F ratio" profile. The result showed that the valley at MHM region still existed (Additional file 8). This argues that at least in gastrulating chicken embryos, dosage compensation is enriched at the MHM region. These results support the existence of temporal differences in the regulation of the compensated and non-compensated genes during embryonic development. Similarly, dosage compensation in the mouse is also a temporal process. In undifferentiated female and male mouse embryonic stem cells and embryonic blastocysts, the F:M ratio of X-linked genes is 1.61. In differentiated cells, the F:M ratio drops to 1.10 [24]. Although the major peak at 6.0 × 10^7 ^bp is observed regardless of tissues types in later stage embryos [8], comparing undifferentiated tissues at gastrulation stage with differentiated somatic and gonadal tissues at E10 and later stages may not be directly relevant. Thus, we cannot rule out that the difference in dosage compensation patterns between gastrulation stage embryos and much later stage embryos is also tissue specific.

To further investigate the behavior of compensated and non-compensated genes, the expression levels of compensated and non-compensated probesets along with probesets on the entire Z chromosome in females and males were plotted. The mean expression levels of compensated and non-compensated genes on the Z chromosome in females were similar. The mean expression level of compensated genes in the male is similar to that of the female. However, the mean expression level of non-compensated genes in males is twice to that in females (Figure 3d). These findings support the conclusion that within the Z chromosome, dosage compensation is achieved through relative downregulation of the expression of the two copies of Z genes in the male, but not through upregulation of a single copy of Z genes in the female.

### Dimorphic expression of genes implicated in sex determination and physiology during early embryonic development

The determinants that control sex determination in birds are unknown [5]. In mammals, the *Sry *gene on the Y chromosome initiates a gene cascade that induces differentiation of the gonads into testes [16]. There is currently no evidence of the existence of an SRY ortholog in the chicken. In our male- and female-biased gene lists, we do not find homologs of other genes involved in the mammalian gonad differentiation pathway, such as: STF1, SOX3, SOX9 or WNT4 [16]. In the chicken, these four genes are not located on the Z chromosome. In humans, loss of a copy of the sex determinant DMRT1 can lead to sex reversal in which a genetically male individual adopts a female phenotype [25]. DMRT1 is Z-linked and sexually dimorphic in the chicken gonad [26,27]. DMRT1 is required for male gonadal sex determination [13]. This gene has homologs in mammals, flies and worms, which are also involved in sex determination [27]. However, in our gene lists, DMRT1 is not significantly male-biased: Gga.260.2.S1_a_at, M:F ratio 1.3, q-value 54%.

The lack of Z chromosome-wide compensation in gastrulating chicken embryos could be explained because male-biased genes were selected to escape compensation in order to serve an active role in male sex determination. In a series of previous studies [7][8][9][10][11], no sex-biased gene was reported to be implicated in sexual determination or differentiation [7][8][9][10][11]. To re-examine this possibility, a gene ontology (GO) analysis test was conducted on the differentially regulated genes between males and females. The quality of the chicken genome GO annotation is extremely poor compared to mouse and human. To circumvent this, we developed an annotation database called Manteia (see Methods section; manuscript in preparation), which links the chicken Affymetrix probesets to the corresponding NCBI or ENSEMBL gene models and merges the available information for both. In addition, Manteia offers the possibility to enhance this annotation by inferring to the chicken gene models the annotation (such as GO terms) of their corresponding human and mouse orthologs. The database provides convenient statistical tools to evaluate the enrichment of GO (or phenotype, see below) categories compared to the genome or to the transcriptome represented on the microarray using an approach originally developed for GoMiner [28].

The GO enrichment analysis tool in Manteia was used to analyze the enrichment in specific GO categories associated with sexual and particularly male differentiation in genes that are expressed significantly higher in males (Additional file 1). The GO enrichment was conducted using the hypergeometric distribution corrected for multiple testing using the Benjamini-Hochberg false discovery rate approach. This analysis identified significant GO terms, such as steroid hormone biosynthesis, steroid hormone receptor activity, spermatid differentiation and androgen receptor signaling pathway enriched in the Z chromosome non-compensated genes (Table 1). The 20 genes belonging to the categories above and to other sex-related GO terms were extracted, 16 of which were on the Z chromosome. From a manual Pubmed search, it was found that ACAA2, AMACR, BBS4, FANCC, FANCG, HEXB, HMGCR, HSD17B4, NR2C1, PAP2a, PELOTA, PLAAP, PRKAA1, PTGER4, SPIN1, StARD4, ZCCHC7, ZCCHC9, DnaJ (Hsp40) homolog subfamily A and C-8 sterol isomerase activity (Table 2) were involved in sex differentiation.

**Table 1:** GO analysis of male-biased genes.

***GO ID***	***Description***	***Raw P-value***	***Adjusted P-value***	***Level***
GO:0006694	steroid biosynthetic process	0.0005	0.01	7
GO:0016126	sterol biosynthetic process	5.712e-05	0.0028	8
GO:0048515	spermatid differentiation	0.06	0.18	7
GO:0030521	Androgen Receptor Signaling Pathway	0.01	0.11	8

Only terms related to sex differentiation and reproduction are included.

**Table 2:** Male-biased genes implicated in steroid metabolism, steroid receptor activity and spermatid and testis development.

***Probeset***	***M:F***	***Gene Title***
Gga.3546.1.S1_at	1.95	hydroxysteroid (17-beta) dehydrogenase 4 (HSD17B4)
Gga.13352.1.S1_at	1.39	zinc finger, CCHC domain containing 7 (ZCCHC7)
GgaAffx.21299.1.S1_at	1.68	TSA*
Gga.15107.1.S1_at	1.43	3-hydroxy-3-methylglutaryl-Coenzyme A reductase (HMGCR)
Gga.17463.1.S1_at	1.45	zinc finger, CCHC domain containing 9 (ZCCHC9)
GgaAffx.11953.1.S1_s_at	1.61	C-8 sterol isomerase activity, Hypothetical protein, clone 7e2Gg-AFFY-23616
Gga.7688.2.S1_s_at	1.79	TSA*
GgaAffx.11719.1.S1_s_at	1.76	acetyl-Coenzyme A acyltransferase 2 (ACAA2)
GgaAffx.9426.1.S1_at	2.26	protein kinase, AMP-activated, alpha 1 catalytic subunit (PRKAA1)
Gga.15316.1.S1_s_at	2.00	StAR-related lipid transfer protein 4 (StARD4)
GgaAffx.12320.1.S1_at	1.75	TSA*
Gga.9708.2.S1_a_at	1.23	Phosphatidate phosphohydrolase type 2a (PAP2a)
Gga.5644.1.S1_s_at	1.38	DnaJ (Hsp40) homolog, subfamily A, member 1RCJMB04
Gga.5644.2.S1_s_at	1.66	TSA*
Gga.7400.1.S1_at	1.62	Bardet-Biedl syndrome 4 protein (BBS4)
Gga.3857.1.S1_s_at	1.73	Fanconi anemia, complementation group G (FANCG)
GgaAffx.8007.1.S1_at	1.73	Fanconi anemia, complementation group C (FANCC)
Gga.12867.1.S1_at	1.74	nuclear receptor subfamily 2, group C, member 1 (NR2C1)
Gga.9970.1.S1_at	1.74	Hexosaminidase B (HEXB)
Gga.4322.1.S1_at	1.76	Spindlin-1 (SPIN1)
GgaAffx.1170.1.S1_at	1.91	Phospholipase A-2-activating protein (PLAAP)
GgaAffx.8764.1.S1_s_at	1.63	pelota homolog (Drosophila) (PELOTA)
GgaAffx.12386.1.S1_at	1.66	TSA*
GgaAffx.9422.1.S1_at	1.61	prostaglandin receptor EP4 subtypeL (PTGER4)
GgaAffx.11974.1.S1_at	1.98	alpha-methylacyl-CoA racemase (AMACR)

TSA*, probeset corresponds to the same gene as the immediate above.

In *Drosophila*, PELOTA mutation causes a male-specific failure of spermatocyte meiosis [29]. In the mouse, Spindlin (SPIN1 homolog) encodes a protein associated with the oocyte meiotic spindle [30], and is highly similar to *Ssty*, a gene on the Y chromosome that is exclusively expressed in spermatids, and which is important for their development [31]. Female chickens also have a SPINW homolog (Gga.1845.1.S1_at) on the W chromosome, but it is not expressed at this stage (data not shown), suggesting that the identified Z-linked SPINZ homolog could, in fact, be related to Ssty. Thus, male-biased expression of Z-linked PELOTA and SPINZ may play a role in spermatocyte development in the chicken.

Strikingly, enzymes involved in steroid synthesis and genes mediating testosterone activity are enriched in the noncompensated, male-specific Z-linked genes. Testosterone, one of the major steroids, is synthesized from cholesterol. We identified several genes involved in cholesterol synthesis - such as ACAA2 (acetyl-Coenzyme A acyltransferase 2) and HMGCR (3-hydroxy-3-methylglutaryl-Coenzyme A reductase), the rate-limiting enzyme in cholesterol synthesis - that are enriched in male-biased genes. StARD4, which is implicated in transporting cholesterol for steroid synthesis; the C-8 sterol isomerase, the prostaglandin receptor EP4, subtype L (PTGER4) and the hydroxysteroid (17-β) dehydrogenase (HSD17B4) are also found in the Z-linked dimorphic genes.

The androgen receptor mediates testosterone or dihydrotestosterone activity and is dimorphically expressed in the adult mouse brain [32]. Nuclear receptor subfamily 2, group C, member 1 (NR2C1), also called testicular receptor 2 and which is normally expressed in testis and prostate in mammals, is acting as a co-receptor of an androgen receptor [33]. It is also listed as a non-compensated Z-linked gene. Additional genes that are involved in the control of reproductive functions (such as, hexosaminidase B [HEXB]), which is involved in the breakdown of gangliosides), are also found in the non-compensated Z-linked genes. In humans, mutations of HEXB cause Sandhoff disease, a progressive neurodegenerative disorder characterized by an accumulation of GM2 gangliosides in neurons. Additionally, HEXB has been shown to have much higher activity in male infants. This bias is correlated with higher testosterone levels in males [34]. This supports the notion that chicken orthologs of these genes are involved in sexual differentiation.

Using the MGI (Mouse Genome Informatics) phenotype descriptions of mouse knockouts in Manteia, the enrichment in specific phenotypes in the mouse orthologs of the non-compensated chicken genes of the Z-chromosome was calculated. Significant enrichment was found in categories, such as seminiferous tubule degeneration (three genes, *p *= 0.002), abnormal seminiferous tubule morphology (six genes, *p *= 0.00091), abnormal testis morphology (eight genes, *p *= 0.00088), abnormal sex gland morphology (eight genes, *p *= 0.0049), abnormal male reproductive anatomy (eight genes, *p *= 0.002), small testis (five genes, *p *= 0.01), abnormal fertility/fecundity (12 genes, *p *= 0.002), reproductive system phenotype (13 genes, *p *= 0.07), and abnormal male reproductive physiology (eight genes, *p *= 0.004). The genes associated with these phenotypes largely overlap the ones identified by GO analysis (Additional file 9).

GO analysis of the 114 compensated Z genes revealed that the major categories enriched include: intracellular signaling cascade (22 genes) or regulation of transcription (21 genes), but do not include significant processes related to sex determination or differentiation (data not shown) [8-10]. Strikingly, the FEM1C gene, which acts downstream of the DMRT1 homolog Mab3 in the *C. elegans *sex determination cascade, belongs to this list of compensated genes [1]. Using the MGI data, enrichment in specific phenotypes for the mouse orthologs of these chicken genes was also analyzed, revealing that 32 of these genes are associated with lethality-embryonic perinatal phenotypes (*p *= 0.0003791394). The most significant phenotypes observed were related to the nervous system and muscle development, but no enrichment for phenotypes related to sexual differentiation was observed (data not shown).

### Early dimorphic expression in the developing embryo of genes implicated in steroid hormone activity and reproduction

**Figure 4:**
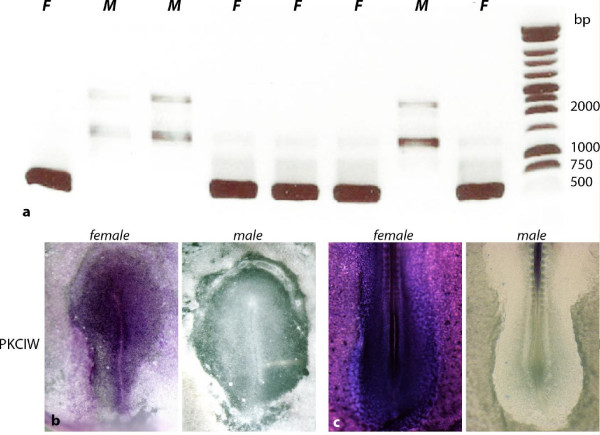
**Female-biased gene expression**. (**a**) The sex of samples can be pre-identified by PKCIW-based PCR genotyping. A PCR product of 500 bp indicates female genotype. F, Female, M, Male. (**b**) and (**c**) PKCIW is expressed in the female but not in the male at HH4 (**b**) and HH15 (**c**). In the male embryo (**c**), the staining in neural tube cavity is non-specific.

**Figure 5:**
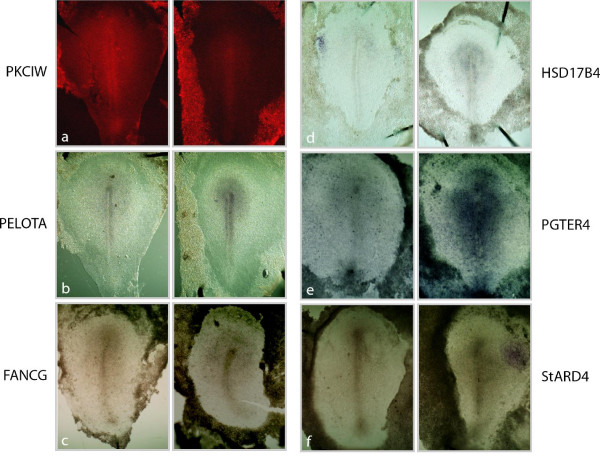
**Male-biased gene expression**. (**a-g**) HH4, dimorphic expression of PKCIW (**a**), PELOTA (**b**), FANCG (**c**), HSD17B4 (**d**), PGTER4 (**e**) and StARD (**f**). (**a**) and (**b**) are the same female and male embryos hybridized by the two different probes. In each panel, the embryo on the left is female and on the right is male.

We performed *in situ *hybridizations to validate some of the female- and male-biased genes. The sex of embryos was ascertained by genotyping with PKCIW-based PCR before processing for *in situ *(Figure 4a). The expression patterns of two female-biased genes, PKCIW and thioredoxin-like 1 (TXNL1) were examined. Consistent with the microarray data and a previous report [15], PKCIW is constitutively expressed in females but not at all in males (Figure 4b and 4c). TXNL1, a Z-linked gene, is expressed 11 times more strongly in females than in males based on microarray analysis (Additional file 2). *In situ *hybridization indicates that it is expressed in females, but is almost undetectable in males (data not shown). To validate genes with an approximate two-fold expression difference between males and females, the following genes - PELOTA, FANCG, HSD17B4, PGTER4 and StARD4 - were randomly chosen to perform double *in situ *hybridization with a PKCIW-specific *in situ *probe. The PKCIW *in situ *signal was revealed first using Tyramide fluorescent substrate (Methods) to ascertain the sex of the embryo (Figure 5a). The stages of male and female samples were carefully identified based on morphology. Then, the *in situ *signals of stage-matched female to stage-matched male embryos were compared (Figure 5b-f) (see Methods). For each gene, four pairs of female/male samples in average were compared. At HH4, the stage when the microarray samples were prepared, both female and male embryos expressed these genes. The expression domains are largely in the anterior half of the embryo proper and are similar in females and males. However, males exhibited an apparent stronger staining (Figure 5b-f). We also analyzed their expression patterns at HH5 and HH6, when the head process and neural plate begin to take shape. In several of the genes, expression levels increase from HH4 to HH5. The dimorphic expression exists at these stages as well (data not shown). Interestingly, although the spatial patterns of most of the genes are somewhat different, the expression was usually highest in the head process and neural plate (data not shown).

## Conclusions

In this report, we used a microarray approach to analyze genes exhibiting sexual dimorphism in the primitive streak of gastrulating male and female chicken embryos, several days before the appearance of gonadal primordia and sexual differentiation. Thus far, the earliest embryos analyzed by this approach were harvested at day 4 [6,7,9], around the beginning of sexual differentiation. Strikingly, we observed that even before the segregation of the three embryonic germ layers, a large number of genes present on the Z chromosome are expressed at a much higher level in males than in females. Since males exhibit two copies of the Z chromosome and females only one, these results are consistent with inefficient dosage compensation of the Z chromosome genes in the chicken [7,8].

During late embryonic stages in mammals, the developing testes secrete two major hormones: anti-Mullerian and testosterone. The anti-Mullerian hormone inhibits the female development of the reproductive tract and external genitalia, while testosterone promotes masculine development [35]. Testis-derived testosterone not only induces male configuration of the reproductive tract and external genitalia, but also circulates to induce permanent changes in discrete nuclei of hypothalamus and other regions of the brain, leading to structural differences and distinct mating behaviors [36]. It is generally believed that testis is the main, if not the only, source of testosterone that acts on the embryonic and adult male brain. This concept is supported by the fact that the brain of castrated male mice is transformed into a female-like brain [37], which highlights the importance of testis and testosterone in the differentiation of dimorphic structures of the brain. In birds, steroid hormones also play important roles in somatic sex dimorphism, especially in the dimorphism of brain regions controlling singing behaviors of song birds, such as zebra finches [38]. However, gonads seem to have little or no role in sex dimorphism in the adult zebra finch brain, since castrated males can still sing male-specific courtship songs [39]. Existence of gynandromorphs in birds clearly supports the notion of sexual dimorphism being largely independent from circulating hormone levels. In some gynandromorph birds, each side of the animal exhibits secondary sexual characteristics, such as feather coloration and patterns that are specific to each sex [40]. In a reported case, one half largely developed like a female, despite the presence of a testis [40].

Our data demonstrate enrichment for genes involved in male sexual differentiation among the non-compensated Z-linked genes. Dimorphic expression of these genes at the gastrulation stage in chicken embryos is striking and can hardly be related to gonadal development, suggesting that, rather, they act on somatic tissues. Interestingly, male zebra finch brain tissue has been shown to be capable of synthesizing estrogen locally. This autonomous synthesis of estrogen is necessary and sufficient for some aspects of male-specific neural development *in vitro *[41,42]. Given the profound effect of testosterone on differentiation of both gonadal and somatic sex, it is tempting to suggest that the chicken may have evolved a mechanism different than mammals to synthesize high levels of testosterone for somatic sex differentiation in males independent of testis formation. Male-biased expression of Z-linked sex differentiation gene expression is likely a result of ineffective dosage compensation.

## Methods

### Sample preparation and microarrays

Fertilized White Leghorn chicken eggs were purchased from commercial sources. Embryos were incubated at 38°C for 23 h to reach HH4-4^+ ^before harvesting. A 400 micron × 400 micron square tissue consisting of the three germ layers was excised at the 70% to 90% primitive streak level of an individual embryo. In this way, the primitive streak proper and epiblast lateral to it were sampled. Thus, this tissue encompasses the prospective somitic mesoderm, neuroectoderm and endoderm. Total RNA was extracted, amplified two cycles and biotin-labeled before being hybridized to Affymetrix Chicken GeneChips. Detailed procedures are essentially the same as previously described [43]. In total, 18 samples were prepared from 18 individual embryos randomly chosen with regard to sex. Sexes were identified by virtue of the presence or absence of high expression of PKCIW genes in 9 of the 18 samples. To monitor the quality of RNA extraction and amplifications during the procedures, some embryos were electoporated with plasmids expressing various constructs, such as the fluorescent reporter Zsgreen. Since no statistical difference was observed in the sexually dimorphic genes between control and electroporated embryos of the same sex, all the samples were subsequently pooled in the analysis. Original microarray data was deposited into NCBI-GEO with access using the following link: http://www.ncbi.nlm.nih.gov/geo/query/acc.cgi?token=xxoffuogsawoavo&acc=GSE16064

### Data processing

The original dataset consisted of 38535 probesets. Since the chicken genome is not very well annotated in terms of the genes corresponding to probesets, and hence to avoid losing information, we decided to treat each probeset as a potential gene. The dataset was normalized by Affymetrix Microarray Suite 5 (MAS5) preprocessing method. To remove the unexpressed genome, the dataset was filtered on the present calls calculated from MAS5. Probesets that were called "Present" in at least one half of the total samples were selected, resulting in 14548 probesets (37% genome). Significance Analysis of Microarrays (SAM) was used to identify differentially regulated genes at a cutoff of a 5% FDR. Applying different thresholds for filtering (Present in one third of the samples, 6 of 18) yielded similar results after SAM analysis. The significance of overlaps between two sets of genes was calculated by defining the appropriate sample space (filtered list of 14548 probesets for differentially expressed genes and Z-chromosome probesets for compensated and non-compensated overlaps) and then randomly selecting the specified number of genes in each group. This was simulated 10000 times to obtain a *P*-value. The comparisons with Melamed and Arnold studies [8] were done using DAVID [44] by extracting the Entrez gene identifiers from the ID conversion interface. The probeset annotation consisted of standard chicken genome annotation as well as from orthology to other organisms (computed by Manteia). Some of the probesets corresponded to 2 or more models of the same gene. This duplication of probeset-gene annotation can result in faulty conclusions in GO analysis. Manteia removes the multiplicity of annotation by assigning a single gene to a probeset by either removing the most poorly annotated genes or choosing one randomly when all are well annotated. In Table 2, when multiple probesets corresponded to a single gene, only the probesets with significant fold change were retained. All statistical analyses and graph generation were conducted using the Bioconductor 2.0 package on R-2.6.1 platform.

### GO analysis

The 406 differentially regulated probesets between males and females corresponding to 246 Ensembl genes were chosen for further analysis. Manteia-GOstat interface http://research.stowers-institute.org:8000/Manteia was used to calculate the significantly enriched GO categories in the dataset. The *P*-values were corrected for multiple tests using FDR [45]. Some of the relevant GO Biological Process (BP) categories along with their adjusted *P*-values are listed in Table 1. Some of the BP categories (such as, "steroid BP" and "Sterol BP") were significantly enriched; whereas, other categories (such as "spermatogenesis") have a *P*-value < 0.2, and thus were not included.

### Cloning and *in situ *hybridization

Genes were cloned by PCR amplification using sequence-specific primers and cDNA prepared from a mixed pool of HH3 to HH6 chicken embryos. To make antisense *in situ *probes, T7 transcription sequence was added to the 3' reverse primer. Antisense *in situ *probes were made using T7 *in vitro *transcription reactions with the PCR products as templates. The primer sequences for the cognitive genes are listed as follows (The T7 promoter sequence is highlighted in bold.):

FANCG: forward (AACTACAGGCACCTTTGCACC); reverse (**CTAATACGACTCACTATAGGG**GGCTATTTCCACTCCTGATCTC).

HSD17B4: forward (GGAGCTTCAGTGGTTGTGAATG); reverse (**CTAATACGACTCACTATAGGG**CTGCTGCGGAGAAACAAAGGAG).

PELOTA: forward (TACCACACCATTGAGCTGGAGC); reverse (**CTAATACGACTCACTATAGGG**TCTGTTTCTGGTACTGCTGGAG).

PGTER4: forward (AAGTCCAGGAAGGAGCAGAAGG); reverse (**CTAATACGACTCACTATAGGG**TTCAATGTCTCAGTGGGGAAGG).

PKCIW: forward (CTGTGAGATACCCACCCTCAG); reverse(**CTAATACGACTCACTATAGGG**AAGCAGTGTCAAAACTCCGAG).

StARD4: forward (ATGCAGTGGTGTGGCGTAAACC); reverse (**CTAATACGACTCACTATAGGG**CCAGAGGAACATCTTCCATGTG).

TXNL1: forward (TGAGTAACAAGTACCCTCAGG); reverse (**CTAATACGACTCACTATAGGG**CACGTAATGGGGATTACAACAG).

To identify the sex of embryos before harvesting for *in situ*, we genotyped embryos using PKCIW 3' UTR-specific, primer-based PCR reactions on genomic DNA. Briefly, a piece of embryonic tissue was extracted and lysed in lysis buffer. 2 μl of crude lysate was added as a template to PCR reaction using the primer pair: forward (CTGTGAGATACCCACCCTCAG)/reverse (TGAGATACCCACCCTCAGTCC). Genomic DNA of female embryos gives rise of a product band of 500 bp, but does not in male embryos. Fluoresceine-labeled antisense riboprobes of genes of interest were synthesized *in vitro*. *In situ *probes were co-hybridized with Digoxigenin-labeled PKCIW probes to HH4-to-HH6 embryos. Alkaline phosphotase conjugated anti-fluoresceine Fab and horseradish peroxidase conjugated anti-Digoxigenin Fab (Roche) were diluted and added together at 1:1000. First, PKCIW *in situ *was revealed as red fluorescence with fluorescent TSA-Cy3 substrate (Perkin Elmer). Then, the sex of samples was identified by the presence or absence of PKCIW *in situ *signal. Samples were then regrouped as females or males. The *in situ *signal of the gene of interest was revealed by adding NBT/BCIP substrate for alkaline phosphotase reaction. Time of coloration was the same for the female group and male groups. Female and male samples of the same gene were photographed at the same illumination and exposure conditions so that *in situ *signals could be directly compared.

## Abbreviations

HH: Hamburger and Hamilton; FDR: false discovery rate; MHM: male hypermethylated region; GO: gene ontology.

## Authors' contributions

OP and SOZ designed the experiments. SOZ performed the experiments and interpreted the results. SM performed the statistical and GO analyses. GH participated in the statistical analysis. OT developed Manteia. OP carried out some of the GO analyses. SOZ, SM and OP wrote the manuscript. All authors read and approved the final manuscript.

## Supplementary Material

Additional file 1 **List of the probesets that are expressed significantly higher in males than in females**. The cutoff is set at 5% FDR. Click here for file

Additional file 2 **List of the probesets that are regarded as male-biased**. The cutoff is set at M:F ratio of 1.5. Click here for file

Additional file 3 **List of the probesets that are expressed significantly higher in females than in males**. The cutoff is set at 5% FDR. Click here for file

Additional file 4 **List of the probesets that are regarded as female-biased**. The cutoff is set at F:M ratio of 1.5. Click here for file

Additional file 5 **List of all the 689 chrZ probesets that are expressed in day-1 embryos**. The M:F ratios of these probesets are calculated. Click here for file

Additional file 6 **List of the probesets that are regarded as compensated**. The criteria is M:F ratio value in the range of 0.8-1.3. Click here for file

Additional file 7 **List of the probesets that are regarded as non-compensated**. The criteria is M:F ratio value greater than 1.5. Click here for file

Additional file 8 **Amplitude map of Z chromosome gene expression**. The running averages of absolute values of log_2 _(M:F) are plotted along Z chromosome position. Click here for file

Additional file 9 **List of mouse genes with orthologs in the list of chicken non-compensated Z genes**. These genes are involved in mouse male reproductive traits as indicated by GO analysis. Click here for file
