# Supplementary figures and images for: Sex-dimorphic gene expression and ineffective dosage compensation of Z-linked genes in gastrulating chicken embryos
Source: BMC Genomics. 2010 Jan 7;11:13. doi: 10.1186/1471-2164-11-13 (PMC2821371; doi:10.1186/1471-2164-11-13)

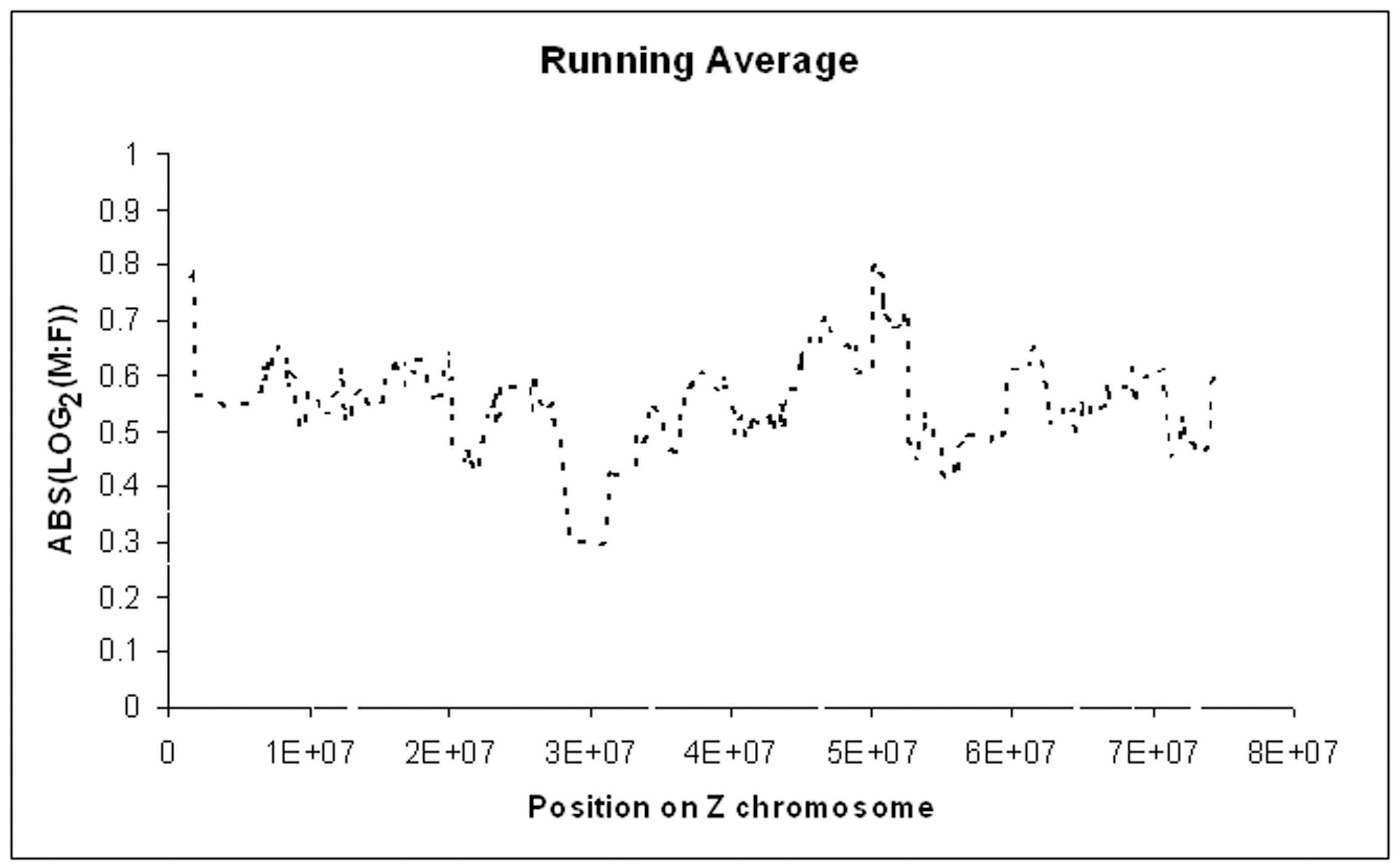

Supplement: Additional file 8 — Amplitude map of Z chromosome gene expression. The running averages of absolute values of log2 (M:F) are plotted along Z chromosome position. [file 1471-2164-11-13-S8.JPEG]
